# BioAgents: Bridging the gap in bioinformatics analysis with multi-agent systems

**DOI:** 10.1038/s41598-025-25919-z

**Published:** 2025-11-07

**Authors:** Nikita Mehandru, Amanda K. Hall, Olesya Melnichenko, Yulia Dubinina, Daniel Tsirulnikov, David Bamman, Ahmed Alaa, Scott Saponas, Venkat S. Malladi

**Affiliations:** 1 University of California, Berkeley, Berkeley, CA USA; 2 Health Futures, Microsoft Research, Redmond, WA USA; 3 School of Information, University of California, Berkeley, Berkeley, CA USA; 4 Bakar Computational Health Sciences Institute, University of California, San Francisco, San Francisco, CA USA; 5 Department of Electrical Engineering and Computer Sciences, University of California, Berkeley, Berkeley, CA USA

**Keywords:** Computational biology and bioinformatics, Computer science

## Abstract

Developing end-to-end bioinformatics workflows is challenging, demanding deep expertise in both genomics and computational techniques. While large language models (LLMs) provide some assistance, they often lack the nuanced guidance required for complex bioinformatics tasks, and are resource-intensive. We thus propose a multi-agent system built on small language models, fine-tuned on bioinformatics data, and enhanced with retrieval augmented generation (RAG). Our system, BioAgents, enables local operation and personalization using proprietary data. We observe performance comparable to human experts on conceptual genomics tasks, and discuss future work to enhance code generation capabilities.

## Introduction

Large language models (LLMs) have been applied to various domain-specific contexts, including scientific discovery in medicine^1–3^, chemistry^4^, and biotechnology^5^. Recent advances in LLMs have spurred their use in bioinformatics^6^, a field encompassing data-intensive tasks such as genome sequencing, protein structure prediction, and pathway analysis. One of the most significant applications has been AlphaFold 3, which uses transformer architecture with triangular attention to predict a protein’s three-dimensional (3-D) structure from amino acid sequences^7^. Other applications include the use of protein language models in transforming amino acids into embeddings^8^.

While LLMs demonstrate impressive capabilities, these models have been found to struggle on complex genomics^9–11^ and bioinformatics code generation^12–14^ tasks. LLMs often face challenges with multi-step biomedical reasoning, especially as task complexity increases. They often require multiple attempts to generate correct solutions^15^, and struggle with integrating knowledge across different tools, data formats, and analysis techniques. This reflects broader challenges in modeling procedural knowledge, where successful outcomes depend on executing sequences of steps that require both domain expertise and computational precision.

**Fig. 1:**
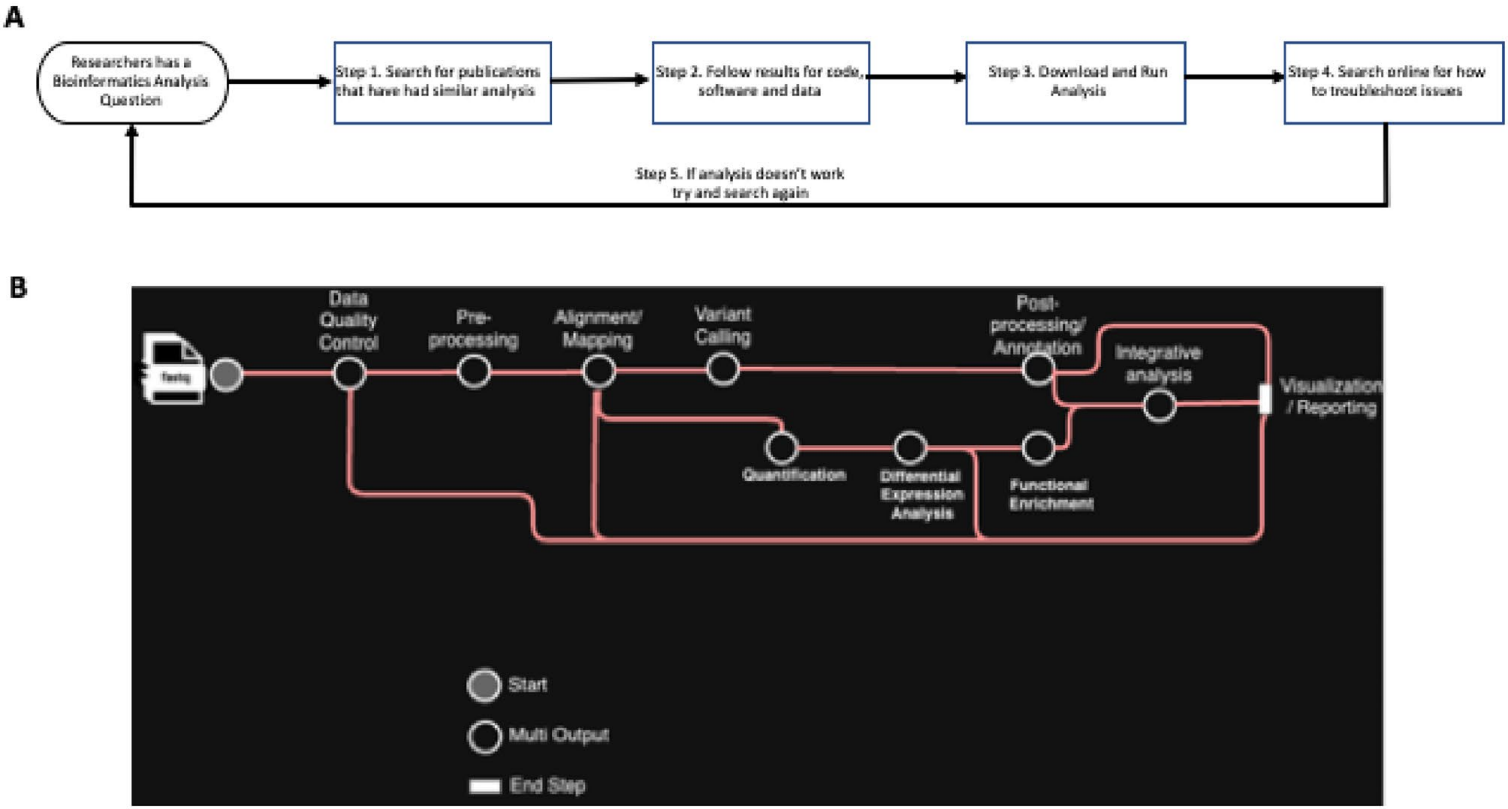
**Supporting Research through Knowledge Graphs and Directed Acyclic Graphs.** (**A**) A knowledge graph showcases the current state-of-the-art for a researcher to start from a research question and navigate through relevant data, tools, and methods to independently run their analysis. (**B**) A typical bioinformatics workflow represented as a workflow diagram, showcasing the intricate dependencies between tasks such as data preprocessing, genome assembly, annotation, and analysis, where each node represents a computational step and edges indicate the flow of data or control.

Modern bioinformatics workflows highlight these limitations vividly, typically requiring complex, multi-stage pipelines that integrate diverse types of data and procedural dependencies, all of which pose significant barriers to automation and hinder clear interpretability. Bioinformaticians often mine question-answer platforms like Biostars for similar problems, search for reproducible scientific workflow examples (e.g., Nextflow, WDL or Snakemake) and software containers (e.g., Biocontainers) on GitHub^16–18^, or refer to the methods sections of recently published papers for code. A typical bioinformatics workflow may involve preparing raw sequencing data, aligning it to a reference genome, and then identifying genetic variants (as shown in Fig. 1). This complexity can present a steep learning curve for newcomers, and poses challenges for bioinformatics experts to stay up-to-date with new techniques^19,20^, as well as with analysis-specific software versions. While established open-source community platforms provide one-off exchanges, they offer limited guidance for researchers trying to develop complex, multi-step workflows across a network of on-premise and cloud infrastructure^21^. As a result, there is a need for interactive and dynamic tools that can offer continuous support.

To tackle this, multi-agent systems have gained traction^22,23^. These systems break down complex tasks into specialized sub-tasks handled by autonomous agents that communicate, coordinate, and integrate their outputs to achieve overarching goals. For example, BioMaster^24^ leverages retrieval-augmented generation (RAG) to dynamically retrieve domain-specific knowledge, improving adaptability to new tools and niche analyses; however, the system primarily focuses on coding and tool execution, which limits its ability to perform higher-level conceptual reasoning, a backbone of complex biomedical workflows.

To bridge this gap and democratize access to bioinformatics knowledge, we introduce BioAgents, a multi-agent system designed to assist users in developing and troubleshooting complex bioinformatics pipelines. Recognizing the potential of multi-agent frameworks^22,23,25,26^, our system provides an interactive solution that adapts to the ongoing needs of users working in specialized domains^27–31^.

**Fig. 2:**
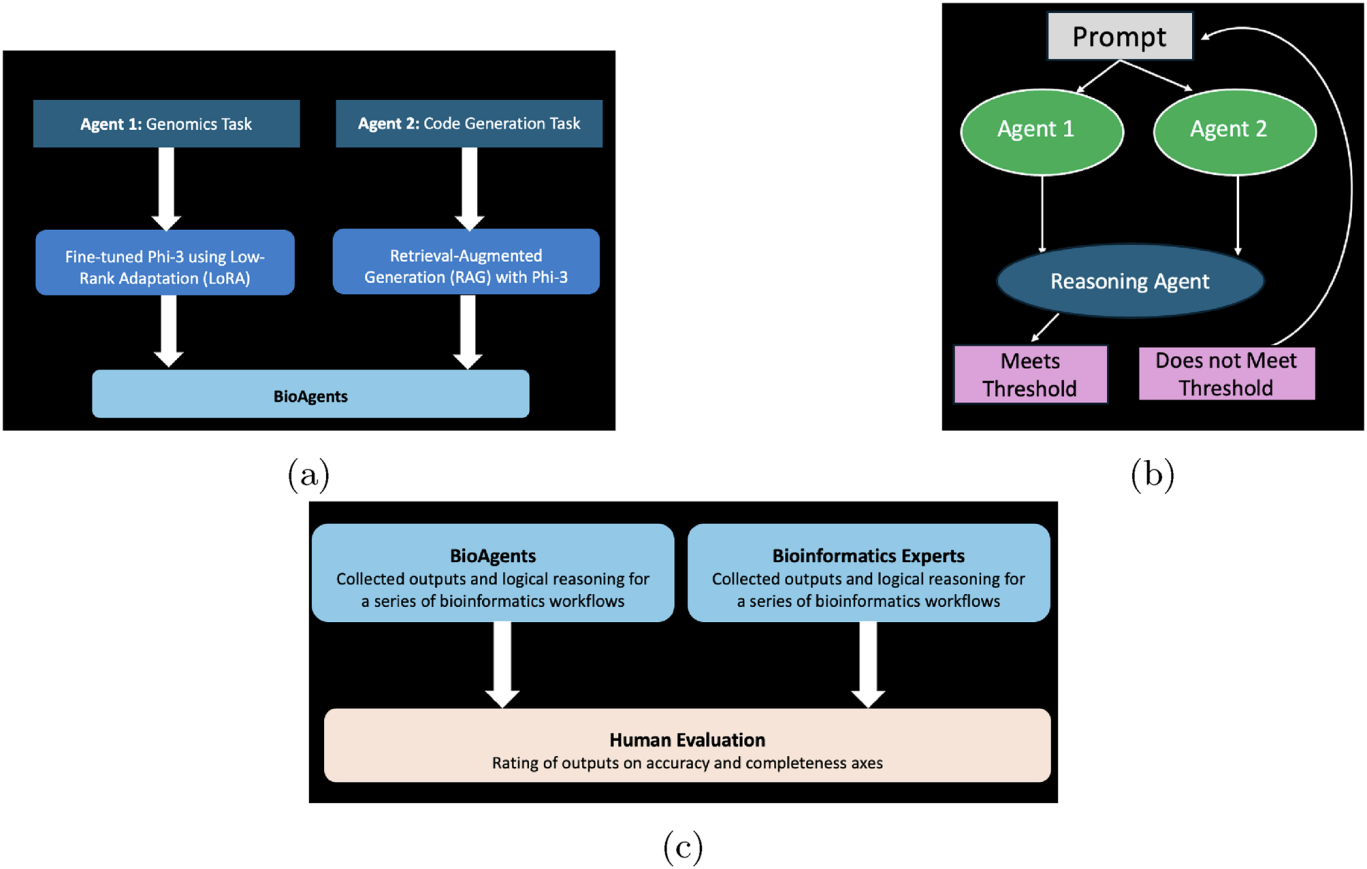
(**a**) **Two Specialized Agents.** Each specialized agent used Phi-3. The first agent focused on conceptual genomics tasks and was fine-tuned on bioinformatics tools documentation, while the second agent used RAG on workflow documentation. (**b**) **Overview of BioAgents.**The reasoning agent, a baseline Phi-3 model, processes the outputs from each specialized agent independently and generates the final response. (**c**) **Comparison of BioAgents’ Outputs with Expert Outputs.**

To better understand the challenges faced by practitioners, we analyzed 68,000 question-answer (QA) pairs from Biostars, extracting the associated tags and categorizing each question. The most frequent questions on the platform revolved around tools, specifically bioinformatics software programs and packages, as well as analysis, such as pipeline-related queries focused on RNA-sequencing, alignment, and variant calling. To address the diverse and complex nature of these questions, we employ multiple specialized agents, each tailored to handle specific tasks such as tool selection, workflow generation, and error troubleshooting, enabling a modular and efficient approach to solving bioinformatics challenges. These insights directly informed the development of our two specialized agents within BioAgents.

While existing multi-agent systems primarily rely on large language models^24,32,33^, we leveraged a smaller, more efficient language model, Phi-3^34^. By using a smaller language model, we are able to maintain high performance while significantly reducing computational resources and infrastructure^35,36^. Avoiding the heavy infrastructure demands associated with larger models, BioAgents is more accessible for local use and efficient real-time applications.

We used the baseline Phi-3 model to build three agents: two specialized agents and Phi-3 as the reasoning agent. Our first agent focused on conceptual genomics tasks, and was fine-tuned on bioinformatics tools documentation from Biocontainers and the software ontology^37,38^. Our second agent used RAG on nf-core documentation and the EDAM ontology^39–41^. Figure 2 shows the creation of our two specialized agents, an overview of the BioAgents, and our experimental design, respectively.

## Results

### Evaluation across several use cases

We devised three use cases of varying difficulty to evaluate our multi-agent system. These workflows, listed below, were designed to assess both conceptual genomics (analysis steps) and code generation tasks. We recruited bioinformatics experts, and provided them with the same inputs used by the multi-agent system. Each workflow involved completing the conceptual genomics and code generation tasks, providing any additional information needed to aid in answering the user query, and explaining the logical reasoning behind the final output.

**Conceptual genomics and code generation tasks**

*Level 1 Tasks (Easy)*

- How would I provide quality metrics on FASTQ files?
- What code or workflow do I need to write to provide quality metrics on FASTQ files?

*Level 2 Tasks (Medium)*

- How do I align RNA-seq data against a human reference genome?
- What code or workflow do I need to write to align RNA-seq data against a human reference genome?

*Level 3 Tasks (Hard)*

- How can I assemble, annotate, and analyze SARS-CoV-2 genomes from sequencing data to identify and characterize different variants of the virus?
- What code or workflow do I need to write to assemble, annotate, and analyze SARS-CoV-2 genomes from sequencing data to identify and characterize different variants of the virus?

**Fig. 3:**
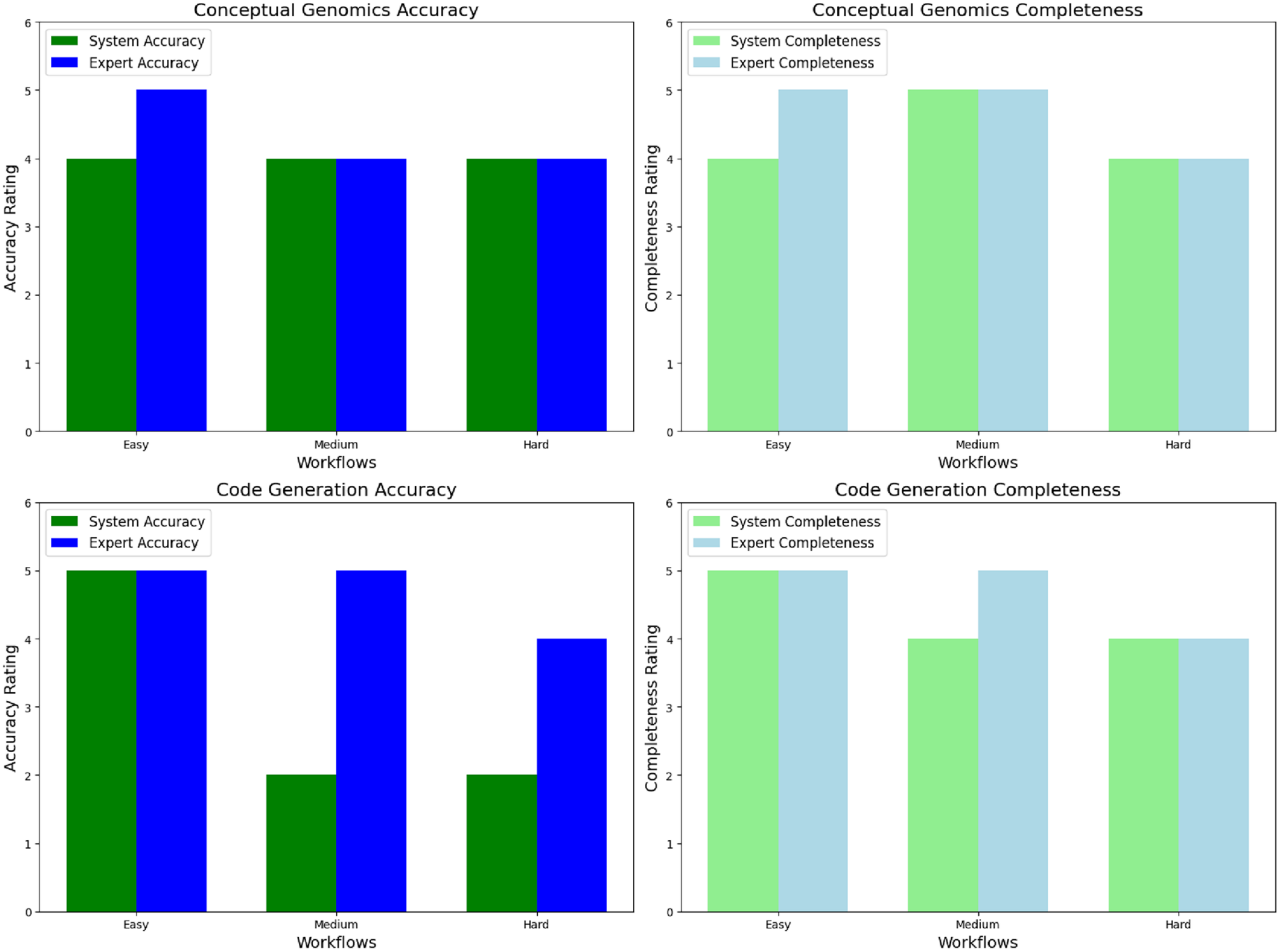
**Comparison of system and expert performance across conceptual genomics and code generation tasks.**The top row evaluates conceptual genomics tasks, with separate panels for accuracy (left) and completeness (right). The bottom row evaluates code generation tasks, similarly split into accuracy (left) and completeness (right). For conceptual genomics tasks, the system demonstrates comparable performance to human experts across all levels of difficulty. In code generation tasks, the system matches expert performance on easier tasks, but shows a decline in accuracy and completeness for medium and hard tasks, highlighting opportunities for improvement in addressing complex challenges.

To assess performance, an expert bioinformatician, part of the team, reviewed both the system and human expert outputs on two axes: 1) accuracy, and 2) completeness. Accuracy was defined as how well the user’s query was answered, while completeness referred to the extent to which the output captured all relevant information in response to the user query. Evaluation results are presented in Fig. 3.

#### Conceptual genomics tasks

BioAgents demonstrated performance on par with experts on conceptual genomics questions across three workflows. This success is largely attributed to our use of Low-Rank Adaptation (LoRA) to fine-tune an agent on the top 50 bioinformatics tools in Biocontainers, including detailed software versions and help documentation. Biocontainers, a widely used bioinformatics service, provides the infrastructure for managing bioinformatics packages and containers, such as conda and docker. Consequently, the system effectively interpreted and responded to these conceptual tasks, achieving human expert-level performance.

**Fig. 4:**
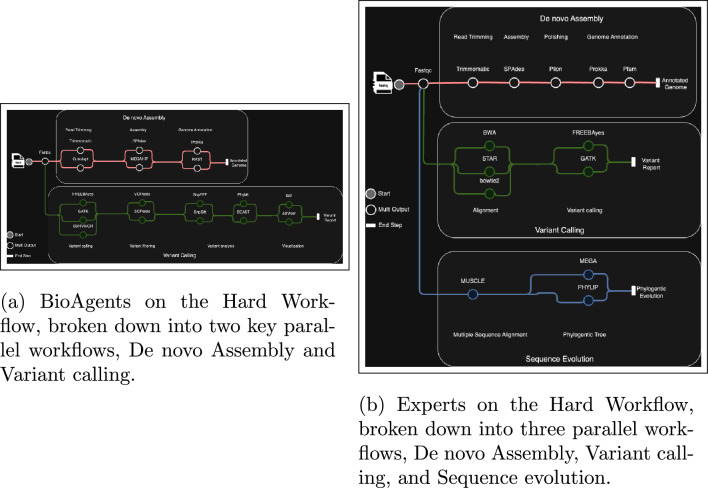
BioAgents and experts on the hard genomics workflow.

In a challenging workflow question on assembling, annotating, and analyzing SARS-CoV-2 genomes from sequencing data, BioAgents provided a logical series of steps, including obtaining sequencing data, performing quality control, assembling the high-quality reads using a de novo assembler, annotating the assembled genome using tools like Prokka or RAST, identifying and characterizing variants, and constructing a phylogenetic tree^42,43^. While human experts proposed robust pipelines, they lacked rationales for tool recommendations. BioAgents did occasionally omit steps though, requiring users to fill in the gaps as shown in Fig. 4.

#### Code generation tasks

Performance discrepancies emerged in code generation tasks, particularly in workflows of increasing complexity. For easy tasks, BioAgents matched expert accuracy, but sometimes provided false information about tools. For medium tasks, representing end-to-end pipelines like those in nf-core workflows (https://nf-co.re/pipelines/), BioAgents struggled to produce complete outputs. In the most complex workflows, the system failed to generate starter code, instead offering step outlines more similar to a conceptual answer. These limitations were attributed to gaps in the indexed workflows, and a lack of tool and language diversity in the training dataset.

### Reliability and transparency

Two key components are necessary in the deployment of multi-agent systems in highly specialized domains: reliability and transparency. Reliability ensures that the system consistently delivers accurate results, while transparency enables users to understand and trust the system’s decision-making process.

#### Self-reflection in agent systems

Several techniques have been proposed to enable a language model to correct its outputs based on internal evaluation, including: self-consistency^44^, self-correction^45,46^, self-evolution^47^, self-feedback^48^ and self-evaluation^49^.

**Fig. 5:**
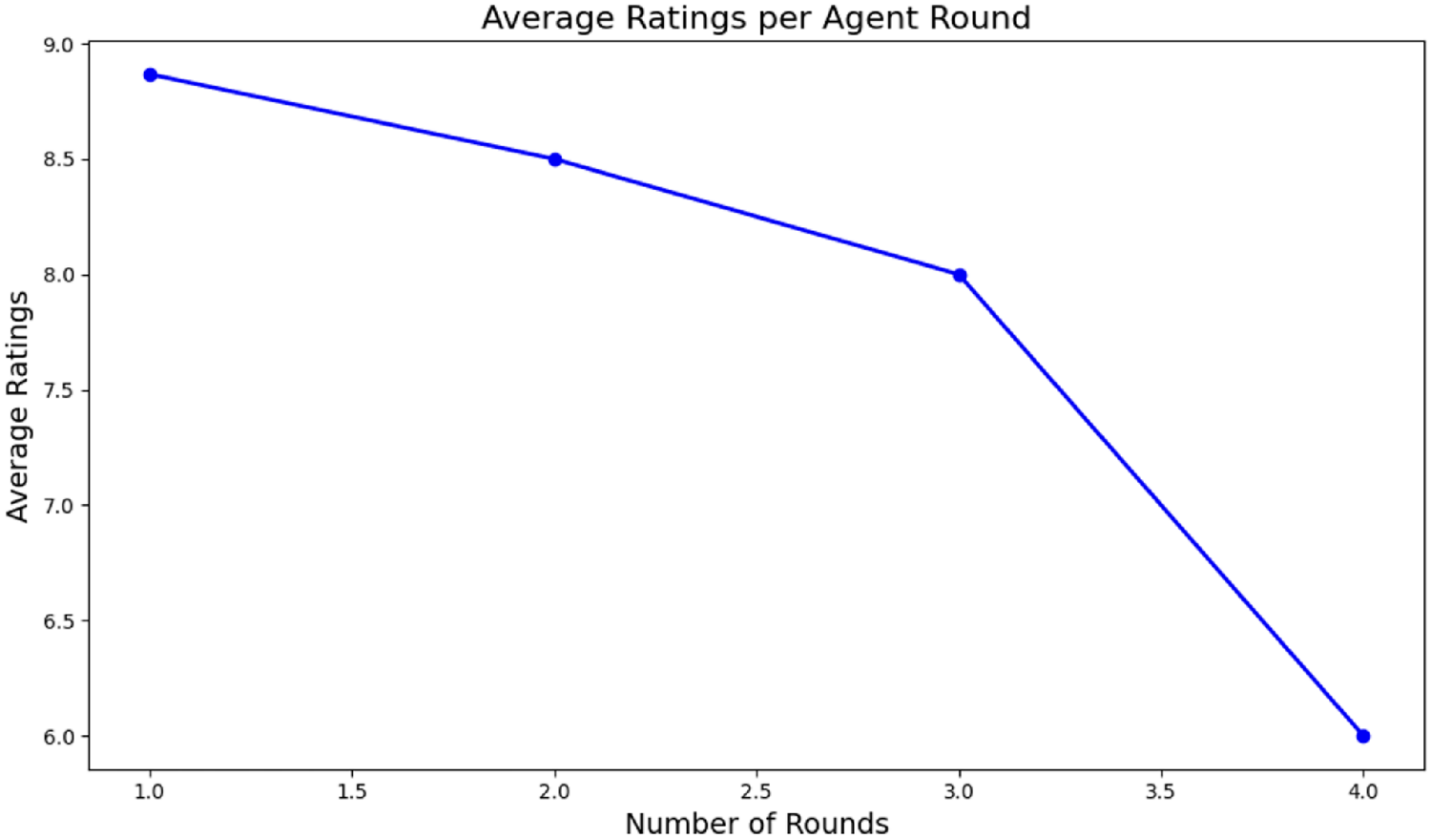
Self-ratings by number of rounds: an inverse correlation between the number of rounds the multi-agent system takes to reach the final answer and the quality of the output’s rating suggests a potential limitation of the iterative processes in multi-agent systems.

BioAgents incorporated self-evaluation to enhance output reliability, inspired by the idea that agent systems can assess the accuracy of their own outputs^50^. Our reasoning agent assessed the quality of responses against a defined threshold. Outputs scoring below this threshold were reprocessed, with agents independently reanalyzing the prompts before returning results. However, the iterative process revealed diminishing returns, where repeated refinements negatively impacted output quality and might not necessarily lead to improved outcomes (Fig. 5).

#### Collaborative reasoning and transparent guidance

Current applications of LLM agents in domain-specific tasks have struggled in the areas of long-term reasoning, decision-making, and instruction-following^51^. Moreover, their proficiency in addressing practical bioinformatics queries and conducting nuanced knowledge inference remains constrained^52^.

In our experimental set-up, both the system and human experts were asked to explain any additional information they would need to better answer users’ questions, and the logical reasoning process behind their answers. Our motivation behind this assessment stemmed from various existing reasoning frameworks – chain-of-thought (CoT)^53^ and ReAct^54^ – used to provide interpretability in LLMs.

In the context of the conceptual genomics medium workflow task, BioAgents explained its rationale for selecting the most suitable RNA-seq alignment tool for mapping against the reference genome, recommending STAR and HISAT2 for their high-throughput and accurate alignments^55,56^. The multi-agent system described how these alignment tools mapped the RNA-seq reads to the reference genome, enabling the identification of genomic locations within the read. BioAgents also specified the factors influencing its choice of alignment tool, noting the size of the RNA-seq dataset and the user’s desired accuracy level as important factors. Generating natural language explanations of model outputs has been shown to improve interpretability, thereby increasing transparency and fostering trust, as users are better able to understand the reasoning behind the model’s specific outputs^57,58^.

A key insight from our findings was that our multi-agent system was able to identify additional information that would have improved responses, even in cases where accuracy was lower. For example, in the hard code generation task, BioAgents struggled to generate the necessary workflow code, but was able to identify additional information that could have better answered the user question, specifically more information on the raw sequencing data, the reference genome sequence, software and tool versions, computational resources (e.g., CPU, memory, disk space), and the user’s bioinformatics experience. In contrast, although the human experts achieved a higher accuracy score on the same task (four compared to two), they described the limitations of their responses, with one human expert noting that the solutions provided were “cobbled together from searching for tutorials,” indicating that it was difficult to identify their information gaps. BioAgents, on the other hand, demonstrated more metacognitive awareness recognizing what it didn’t know, and more importantly, additional information it could benefit from^59^. A consistent theme across the human expert responses was an inability to articulate what additional information they would need to improve their answers. This highlights how vast and expansive of a knowledge base is needed to fully answer these questions, which often extends beyond the expertise of one individual.

## Discussion

The reproducibility crisis in computational research highlights the urgent need for systems that can reliably extract, reproduce, and adapt research findings. This challenge is especially pronounced in bioinformatics, where complex workflows often hinder replication and validation efforts. BioAgents, a multi-agent system, offers a promising solution by potentially extracting methods from research papers, generating executable workflows, and integrating human-in-the-loop approaches to improve accuracy and customization.

BioAgents has the potential to facilitate reproducibility by automatically synthesizing workflows from research publications, enabling researchers to replicate experiments, validate results, and adapt analyses to their datasets. By prioritizing transparency and integrating human feedback, BioAgents ensures outputs are both reliable and user-specific. Furthermore, BioAgents can be extended to clinical settings and other scientific domains. In medicine, the system could assist in replicating diagnostic workflows, personalizing treatment recommendations based on patient data, and optimizing clinical trial designs, ultimately enabling more efficient and reliable translational research. In chemistry and physics, BioAgents can automate the replication of experimental protocols and model complex systems, enhancing the reproducibility of results across a wide range of scientific fields.

Collaborative reasoning highlighted key areas for improvement in reasoning, decision-making, and instruction-following. BioAgents effectively identified information gaps, such as tool versions and user experience, which experts often overlooked. Generating natural language explanations of its decisions increased interpretability, fostering user trust. Despite struggles with accuracy in complex tasks, BioAgents demonstrated metacognitive awareness, outlining additional data that could improve results.

The increased reliability, transparency, and trust fostered by BioAgents is particularly valuable for reducing barriers for new bioinformatics researchers. Unlike static question-answer forums, which can provide solutions without clear explanations or insight into the respondent’s reasoning process, BioAgents allows users to not only receive answers, but also provides the underlying supporting information that led to those answers. By sharing the multi-agent system’s reasoning behind its proposed steps, researchers can learn how to replicate those decision-making processes, highlighting the educational value of our approach. Ultimately, the transparency provided by BioAgents not only improves trust in agentic systems but also facilitates knowledge transfer, allowing users to grow and develop their expertise.

In conceptual genomics tasks, BioAgents demonstrated performance comparable to that of human experts, successfully addressing domain-specific challenges. However, areas for improvement were identified in code generation. Specifically:

- *Workflow Scope*: The system’s reliance on nf-core workflows limited diversity. Expanding indexed workflows to include additional sources could address this gap.
- *Information Retrieval*: Retrieving multiple document matches, rather than relying solely on top-ranked results, could enhance the relevance of generated workflows.
- *Reasoning Agent*: Enhancing the reasoning agent to verify tool versions, ensure executability, and reference source documentation could increase transparency and foster user trust.

### Limitations and future work

We acknowledge that the term “bioinformatics” is very broad, encompassing a wide range of research areas and tasks. A limitation of this study is its narrow evaluation on only three genomics analysis tasks, which represent a small subset of bioinformatics research. A future goal is to expand the diversity of questions to incorporate additional research areas such as image analysis, sequence analysis, multivariate data analysis, and microbiome analysis. This expansion would provide a more robust evaluation of the system’s potential, and its applicability across the broader bioinformatics landscape.

Additionally, the current system is built around a singular small model, Phi-3. Since its initial development and evaluation, many specialized models for coding and reasoning, including o3-mini and Claude Sonnet 4, have been released. Recent work has also argued that small language models (SLMs) are not only sufficiently capable for specialized agentic systems, but inherently more efficient and economical than larger LLMs^60^. To further evaluate our multi-agent model, we plan to substitute Phi-3 with these other models. This will allow us to assess how each performs within different components of the multi-agent system, such as in coding or reasoning tasks. As we change the models in the system, we would also like to evaluate the multi-agent system against either RAG or fine-tuned only systems.

## Conclusion

One of BioAgents’ key strengths is its ability to support user learning. By linking generated workflows to source documentation and providing support information for each step, BioAgents enables researchers to understand and modify workflows, thereby contributing to their professional development and the broader bioinformatics community. Additionally, when BioAgents requests supplementary information, researchers gain the opportunity to refine results tailored to their specific analyses, while also enhancing their understanding of their own data.

By lowering barriers to compute resources and operating seamlessly in local environments, BioAgents addresses both accessibility and scalability. Its potential extends beyond bioinformatics, offering a model for intelligent systems in other domains facing reproducibility challenges to use the BioAgents framework and train on their own propriety or domain-specific code and documentation.

With targeted enhancements in workflow diversity, retrieval methods, and reasoning capabilities, BioAgents is poised to become a cornerstone in the push for reproducible, transparent, and accessible computational research.

## Methods

### Datasets

#### Biostars

Biostars^18^ is an online community platform for the bioinformatics community where users can ask and answer questions related to computational genomics and biological data analysis. We scraped all publicly available data from the site, which included a total of 68,000 question-answer (QA) pairs, up to May 1, 2024. Only answers with at least one user upvote were added to the QA dataset. Tags assigned to each question were extracted, then GPT-3.5 was used to categorize them into one of five categories:

- tool – software programs and packages used for bioinformatics analysis
- analysis – pipelines and analysis performed in bioinformatics field, such as RNA-seq, alignment, variant calling
- data format – genomics and other -omics data formats
- programming – programming languages, including wdl, nextflow and snakemake, and operation systems
- other – for everything else.

We used 71 QA pairs for tool and analysis categories to perform benchmarks across different models (Supplemental Material Table 1).

#### Biocontainers

We fine-tuned BioAgents on Biocontainers’ (https://biocontainers.pro/) top 50 tools Supplemental Table 2, including versions and documentation. We use the TRS API (https://api.biocontainers.pro/ga4gh/trs/v2/ui/) to pull statistics on the top bioinformatics tools, based on download frequency. To get the top 50 tools we cross-referenced the tool statistics with mentions in BioStars data. Then, we grabbed each available docker version of each of those tools. For each docker container, we downloaded the container and outputted command-line help documentation.

#### Ontologies

We downloaded both the Software and EDAM Ontologies^38,39,41^ for software and assay description. To convert to JSON-LD we used the JSON or OBO format to extract the name and either the description or definition .

### Models

We leveraged a single A100 GPU to perform parameter-efficient fine-tuning (PEFT) on the Phi-3-mini-128-instruct model, optimizing it for bioinformatics tasks. Specifically, we employ the QLoRA technique, which enables fine-tuning with reduced computational overhead by quantizing the model’s layers and training low-rank adapters. This approach is particularly well-suited for large-scale language models like Phi-3-mini, as it retains performance while significantly reducing resource requirements.

Our fine-tuning dataset focuses on the top 50 most commonly used BioContainers tools, along with their associated versions and help documentation, ensuring broad applicability to bioinformatics workflows. Additionally, we added Software Ontology data about the name of the software and its purpose. Training was conducted on Azure Machine Learning, with model configurations limited to a new token count of 1,000 and a temperature of 0.1 to control response diversity and precision.

For our RAG implementation, we integrate OpenAI’s text-embedding-ada-002 for high-quality semantic search. The embeddings are indexed within Azure AI’s search service, optimized to retrieve nf-core modules efficiently, and the Sequence Ontology, describing each assay and its purpose. This combination ensures that the system can provide relevant, tool-specific code generation and guidance tailored to bioinformatics workflows.

By combining QLoRA-based fine-tuning and RAG, we achieve a system that balances computational efficiency, domain specificity, and accessibility for researchers in bioinformatics.

### Expert survey

We surveyed expert bioinformaticians on workflow questions derived from Biostars data, comparing their reasoning and logic against outputs from BioAgents. This study component allowed us to evaluate and compare the decision-making behavior of BioAgents with human experts, aiming to enhance state-of-the-art human interpretability in multi-agent AI systems^61,62^. Below we discuss our survey design, respondent recruitment, informed consent process, survey data analysis, and findings.

#### Survey design

We created a survey using Microsoft Forms to obtain human domain expert answers and their reasoning behind responses related to translating genomics tasks, and writing subsequent code to analyze data question types. Biostars community forum questions were abstracted and categorized to evaluate common question types, which we found to be around tools and/or analysis. We then created three levels of questions (easy, medium, and hard) increasing in complexity (i.e., number of steps and knowledge required) derived from questions in Biostars.

The survey consisted of 27 questions. We asked eight demographic questions to assess respondent’s education-level, number of years working with bioinformatics tools, data types they worked with regularly, work setting, programming experience, age, gender, and if English was their first language. For the remaining survey questions, respondents were asked to imagine an undergraduate student asked for help on a set of questions, and to provide the logic and steps they would advise the student to take to successfully complete their inquiry. Each of the three question levels had two parts: (1) answering the question asked by the student with corresponding logic/ reasoning, and (2) generating code (in their preferred programming language) with corresponding logic /reasoning. An optional question was asked after each of these questions around what additional information, if any, they would need to answer the student’s question.

#### Recruitment and informed consent

We recruited survey respondents through a combination of Microsoft internal community distribution email lists and snowball sampling techniques in August of 2024. The primary inclusion criteria for participation required individuals to have 5+ years of bioinformatics experience with tools/ workflows, intermediate to advanced coding skills, and be at least 18 years of age. Our goal was to recruit five to eight bioinformaticians to capture a range of domain expertise in biomedical informatics and to ensure a diverse group of experts.

Five bioinformatician experts responded to our survey. The expert respondents ranged in age (25–44 years), gender (2 women, 3 men), and education level (2 masters, 3 doctorates). All respondents reported intermediate to advanced programming experience, 5 or more years of experience working with bioinformatics tools, and English as their first language. Additionally, all respondents reported working with a range of biomedical data types (3 non-human, 3 human, non-clinical, and 4 human, clinical data) and most reported working in an industry setting.

Respondents were excluded if they had higher executive roles in industry to avoid any potential bias. Respondents interested in the study were sent the Microsoft Forms survey to assess their eligibility based on the study criteria, and to review the study informed consent. If they met the eligibility criteria requirements and agreed to the terms outlined in the informed consent, they proceeded to respond to the survey questions. Respondents were compensated $50 USD for their time via a gift card. This study was reviewed and approval by Microsoft Research Institutional Review Board (ID10950). Written informed consent was obtained from each participant prior to taking the survey. All interviews were conducted in accordance with relevant guidelines and regulations.

#### Qualitative data analysis

We conducted a content and thematic analysis based on the five expert responses to the easy, medium, and hard genomics workflows and code generation task questions. A.K.H. conducted a content analysis of the survey responses to curate a human expert heuristic ground-truth dataset. For the logic/reasoning and additional information needed questions, A.K.H., N.M., and V.S.M. conducted a thematic analysis of emerging themes to compare similarities and differences within and across the expert responses.

#### Expert findings

We aggregated survey responses from the five human expert respondents on conceptual genomics and code generation tasks across the three levels of difficulty (Fig. 2). We created a master list of steps by task, and corresponding code as ground-truth data across all experts’ responses to the genomics workflow and code generation task questions. We took the list of tasks to generate a knowledge graph, represented as a DAG. These DAGs were then used to conduct a thematic analysis for emerging themes based on responses to the logic/reasoning and additional information needed questions.

Human expert responses to questions and corresponding logic plus additional information required did not vary across experts on the easy question types (Supplemental Figs. S1 and S2). However, for the medium (Supplemental Figs. S3 and S4) and hard question (Fig. 4 sets, experts required more logic/reasoning and additional information due to the increased complexity (e.g., number of steps) of the biomedical research tasks necessary to correctly answer the question. This also included the tools and knowledge needed to navigate and implement the correct solution.

### Human evaluation

To compare the performance of the BioAgents system outputs with the responses of the surveyed human experts, V.S.M. scored the outputs and responses based on two key criteria: *accuracy* and *completeness*. For the conceptual task questions, V.S.M assessed whether the system’s reasoning and recommendations were consistent with domain knowledge. In the code generation task questions, the surveyed human experts’ responses were assessed for correctness of syntax, compatibility with relevant tools or environments, and whether the code performed the intended functionality as expected. Each criteria was scored on Likert scale (1 to 5)^63^. We designed a scoring sheet, describing how to differentiate the scores, with specific definitions for each level.

1. *Accuracy (1–5):* The degree to which the output code and steps are correct. The steps and/or software are reasonable and not hallucinated. Where 1 indicates major inaccuracies and 5 indicates full correctness.
2. *Completeness (1–5):* The extent to which the output provides all necessary components or steps required for the task, where 1 indicates significant omissions and 5 indicates comprehensive coverage of code or steps such that the user would be able to implement the answer without searching for additional information.

## Supplementary Information

Supplementary Information 1.

Supplementary Information 2.

## Data Availability

The datasets generated during and/or analysed during the current study are available from the corresponding author on reasonable request.

## Code availability

The complete source code of Bioagents is available online at GitHub: https://github.com/microsoft/bioinformagus.

## References

[1] Singhal, K. et al. Large language models encode clinical knowledge. *Nature* **620**(7972), 172–180 (2023). 37438534 10.1038/s41586-023-06291-2 PMC10396962

[2] Thirunavukarasu, A. J. et al. Large language models in medicine. *Nat. Med.* **29**(8), 1930–1940 (2023). 37460753 10.1038/s41591-023-02448-8

[3] Yang, R. et al. Large language models in health care: Development, applications, and challenges. *Health Care Sci.* **2**(4), 255–263 (2023). 38939520 10.1002/hcs2.61 PMC11080827

[4] Boiko, D. A., MacKnight, R., Kline, B. & Gomes, G. Autonomous chemical research with large language models. *Nature* **624**(7992), 570–578 (2023). 38123806 10.1038/s41586-023-06792-0 PMC10733136

[5] Madani, A. et al. Large language models generate functional protein sequences across diverse families. *Nat. Biotechnol.* **41**(8), 1099–1106 (2023). 36702895 10.1038/s41587-022-01618-2 PMC10400306

[6] Cheng, W., Shen, J., Khodak, M., Ma, J., & Talwalkar, A. L2g: Repurposing language models for genomics tasks. *bioRxiv* 2024–12. (2024).

[7] Abramson, J., Adler, J., Dunger, J., Evans, R., Green, T., Pritzel, A., Ronneberger, O., Willmore, L., Ballard, A.J., Bambrick, J., et al. Accurate structure prediction of biomolecular interactions with alphafold 3. *Nature* 1–3. (2024). 10.1038/s41586-024-07487-w PMC11168924 38718835

[8] Yin, H., Gu, Z., Wang, F., Abuduhaibaier, Y., Zhu, Y., Tu, X., Hua, X.-S., Luo, X., & Sun, Y. An evaluation of large language models in bioinformatics research. https://arXiv.org/abs/2402.13714. (2024).

[9] Bhardwaj, S., & Hasija, Y. Chatgpt, a powerful language model and its potential uses in bioinformatics. In *2023 14th International Conference on Computing Communication and Networking Technologies (ICCCNT)* 1–6. (IEEE, 2023).

[10] Lubiana, T. et al. quick tips for harnessing the power of chatgpt in computational biology. *PLoS Comput. Biol.* **19**(8), e1011319 (2023). 37561669 10.1371/journal.pcbi.1011319 PMC10414555

[11] Sarwal, V., Munteanu, V., Suhodolschi, T., Ciorba, D., Eskin, E., Wang, W., & Mangul, S. Biollmbench: A comprehensive benchmarking of large language models in bioinformatics. *bioRxiv* 2023–12. (2023)

[12] Kang, K., Yang, Y., Wu, Y., & Luo, R. Integrating large language models in bioinformatics education for medical students: Opportunities and challenges. *Ann. Biomed. Eng.* 1–5. (2024). 10.1007/s10439-024-03554-5 38839663

[13] Tang, X. et al. Biocoder: A benchmark for bioinformatics code generation with large language models. *Bioinformatics* **40**, i266–i276 (2024). 38940140 10.1093/bioinformatics/btae230 PMC11211839

[14] Wang, Z., Zhou, Z., Song, D., Huang, Y., Chen, S., Ma, L., & Zhang, T. Where do large language models fail when generating code? https://arXiv.org/abs/2406.08731. (2024).

[15] Piccolo, S.R., Denny, P., Luxton-Reilly, A., Payne, S., & Ridge, P.G. Many bioinformatics programming tasks can be automated with chatgpt. https://arXiv.org/abs/2303.13528. (2023). 10.1371/journal.pcbi.1011511 PMC10564134 37769024

[16] Di Tommaso, P. et al. Nextflow enables reproducible computational workflows. *Nat. Biotechnol.* **35**(4), 316–319 (2017). 28398311 10.1038/nbt.3820

[17] Gruening, B., Sallou, O., Moreno, P., da Veiga Leprevost, F., Ménager, H., Søndergaard, D., Röst, H., Sachsenberg, T., O’connor, B., Madeira, F., et al. Recommendations for the packaging and containerizing of bioinformatics software. *F1000Research 7* ISCB–Comm. (2019). 10.12688/f1000research.15140.1 PMC6738188 31543945

[18] Parnell, L. D. et al. Biostar: An online question & answer resource for the bioinformatics community. *PLoS Comput. Biol.* **7**(10), e1002216 (2011). 22046109 10.1371/journal.pcbi.1002216 PMC3203049

[19] Chatterjee, A., Ahn, A., Rodger, E. J., Stockwell, P.A., & Eccles, M.R. A guide for designing and analyzing rna-seq data. *Gene Expression Anal. Methods Protoc.* (2018), 35–80. 10.1007/978-1-4939-7834-2_3 29767357

[20] Shue, E. et al. Empowering beginners in bioinformatics with chatgpt. *Quantit. Biol.* **11**(2), 105–108 (2023). 10.15302/J-QB-023-0327 PMC10299548 37378043

[21] Patel, K. A beginner’s guide to bioinformatics. *Biochemist* **45**(2), 11–15 (2023).

[22] Li, G., Hammoud, H., Itani, H., Khizbullin, D. & Ghanem, B. Camel: Communicative agents for “mind’’ exploration of large language model society. *Adv. Neural. Inf. Process. Syst.* **36**, 51991–52008 (2023).

[23] Wu, Q., Bansal, G., Zhang, J., Wu, Y., Zhang, S., Zhu, E., Li, B., Jiang, L., Zhang, X., & Wang, C. Autogen: Enabling next-gen llm applications via multi-agent conversation framework. https://arXiv.org/abs/2308.08155. (2023).

[24] Su, H., Long, W., & Zhang, Y. Biomaster: Multi-agent system for automated bioinformatics analysis workflow. *bioRxiv*, 2025–01. (2025). 10.1016/j.patter.2026.101611 PMC13494596 42630761

[25] Singh, A., Ehtesham, A., Mahmud, S., & Kim, J.-H. Revolutionizing mental health care through langchain: A journey with a large language model. In *2024 IEEE 14th Annual Computing and Communication Workshop and Conference (CCWC)* 0073–0078. (IEEE, 2024).

[26] Sreedhar, K., & Chilton, L. Simulating human strategic behavior: Comparing single and multi-agent llms. https://arXiv.org/abs/2402.08189. (2024).

[27] Gao, S. et al. Empowering biomedical discovery with ai agents. *Cell* **187**(22), 6125–6151 (2024). 39486399 10.1016/j.cell.2024.09.022

[28] Mehandru, N. et al. Evaluating large language models as agents in the clinic. *NPJ Digit. Med.* **7**(1), 84 (2024). 38570554 10.1038/s41746-024-01083-y PMC10991271

[29] Wang, L. et al. A survey on large language model based autonomous agents. *Front. Comp. Sci.* **18**(6), 186345 (2024).

[30] Xiao, Y., Liu, J., Zheng, Y., Xie, X., Hao, J., Li, M., Wang, R., Ni, F., Li, Y., Luo, J., et al. Cellagent: An llm-driven multi-agent framework for automated single-cell data analysis. *bioRxiv* 2024–05. (2024).

[31] Xin, Q., Kong, Q., Ji, H., Shen, Y., Liu, Y., Sun, Y., Zhang, Z., Li, Z., Xia, X., Deng, B., et al. Bioinformatics agent (bia): Unleashing the power of large language models to reshape bioinformatics workflow. *bioRxiv* 2024–05. (2024).

[32] Guo, T. et al. What can large language models do in chemistry? A comprehensive benchmark on eight tasks. *Adv. Neural. Inf. Process. Syst.* **36**, 59662–59688 (2023).

[33] M. Bran, A., Cox, S., Schilter, O., Baldassari, C., White, A. D., & Schwaller, P. Augmenting large language models with chemistry tools. *Nat. Mach. Intell.* 1–11. (2024). 10.1038/s42256-024-00832-8 PMC11116106 38799228

[34] Abdin, M., Jacobs, S.A., Awan, A.A., Aneja, J., Awadallah, A., Awadalla, H., Bach, N., Bahree, A., Bakhtiari, A., Behl, H., et al. Phi-3 technical report: A highly capable language model locally on your phone. https://arXiv.org/abs/2404.14219. (2024).

[35] Chen, L., & Varoquaux, G. What is the role of small models in the llm era: A survey. https://arXiv.org/abs/2409.06857. (2024).

[36] Schick, T., Dwivedi-Yu, J., Dessì, R., Raileanu, R., Lomeli, M., Hambro, E., Zettlemoyer, L., Cancedda, N., & Scialom, T. Toolformer: Language models can teach themselves to use tools. *Adv. Neural Inf. Process. Syst.* **36** (2024).

[37] da Veiga Leprevost, F. et al. Biocontainers: An open-source and community-driven framework for software standardization. *Bioinformatics* **33**(16), 2580–2582 (2017). 28379341 10.1093/bioinformatics/btx192 PMC5870671

[38] Malone, J. et al. The software ontology (swo): A resource for reproducibility in biomedical data analysis, curation and digital preservation. *J. Biomed. Semant.* **5**, 1–13 (2014). 10.1186/2041-1480-5-25 PMC4098953 25068035

[39] Black, M., Lamothe, L., Eldakroury, H., et al. Edam: the bioscientific data analysis ontology (update 2021). ISCB Comm J, poster, version 1; not peer reviewed. (2022).

[40] Ewels, P. A. et al. The nf-core framework for community-curated bioinformatics pipelines. *Nat. Biotechnol.* **38**(3), 276–278 (2020). 32055031 10.1038/s41587-020-0439-x

[41] Ison, J., Kalaš, M., Ménager, H., Willighagen, E., Grüning, B., and Ignard, A. edamontology/edamontology: Edam 1.25, (2020).

[42] Aziz, R. K. et al. The rast server: Rapid annotations using subsystems technology. *BMC Genom.* **9**, 1–15 (2008). 10.1186/1471-2164-9-75 PMC2265698 18261238

[43] Seemann, T. Prokka: rapid prokaryotic genome annotation. *Bioinformatics* **30**(14), 2068–2069 (2014). 24642063 10.1093/bioinformatics/btu153

[44] Chen, A., Phang, J., Parrish, A., Padmakumar, V., Zhao, C., Bowman, S.R., & Cho, K. Two failures of self-consistency in the multi-step reasoning of llms. https://arXiv.org/abs/2305.14279. (2023).

[45] Kamoi, R., Zhang, Y., Zhang, N., Han, J. & Zhang, R. When can llms actually correct their own mistakes? A critical survey of self-correction of llms. *Trans. Assoc. Computat. Linguist.* **12**, 1417–1440 (2024).

[46] Pan, L., Saxon, M., Xu, W., Nathani, D., Wang, X., & Wang, W. Y. Automatically correcting large language models: Surveying the landscape of diverse self-correction strategies. https://arXiv.org/abs/2308.03188. (2023).

[47] Tao, Z., Lin, T.-E., Chen, X., Li, H., Wu, Y., Li, Y., Jin, Z., Huang, F., Tao, D., and Zhou, J. A survey on self-evolution of large language models. https://arXiv.org/abs/2404.14387. (2024).

[48] Liang, X., Song, S., Zheng, Z., Wang, H., Yu, Q., Li, X., Li, R.-H., Wang, Y., Wang, Z., Xiong, F., et al. Internal consistency and self-feedback in large language models: A survey. https://arXiv.org/abs/2407.14507. (2024).

[49] Ren, J., Zhao, Y., Vu, T., Liu, P.J., & Lakshminarayanan, B. Self-evaluation improves selective generation in large language models. In *Proceedings on* 49–64. (PMLR, 2023).

[50] Zhuge, M., Zhao, C., Ashley, D., Wang, W., Khizbullin, D., Xiong, Y., Liu, Z., Chang, E., Krishnamoorthi, R., Tian, Y., et al. Agent-as-a-judge: Evaluate agents with agents. https://arXiv.org/abs/2410.10934. (2024).

[51] Liu, X., Yu, H., Zhang, H., Xu, Y., Lei, X., Lai, H., Gu, Y., Ding, H., Men, K., Yang, K., et al. Agentbench: Evaluating llms as agents. https://arXiv.org/abs/2308.03688. (2023).

[52] Chen, Q., & Deng, C. Bioinfo-bench: A simple benchmark framework for llm bioinformatics skills evaluation. *bioRxiv* 2023–10. (2023).

[53] Wei, J. et al. Chain-of-thought prompting elicits reasoning in large language models. *Adv. Neural. Inf. Process. Syst.* **35**, 24824–24837 (2022).

[54] Yao, S., Zhao, J., Yu, D., Du, N., Shafran, I., Narasimhan, K., & Cao, Y. React: Synergizing reasoning and acting in language models. https://arXiv.org/abs/2210.03629. (2022).

[55] Dobin, A. et al. Star: Ultrafast universal rna-seq aligner. *Bioinformatics* **29**(1), 15–21 (2013) (**Epub 2012 Oct 25**). 23104886 10.1093/bioinformatics/bts635 PMC3530905

[56] Kim, D., Paggi, J. M., Park, C., Bennett, C. & Salzberg, S. L. Graph-based genome alignment and genotyping with hisat2 and hisat-genotype. *Nat. Biotechnol.* **37**(8), 907–915 (2019). 31375807 10.1038/s41587-019-0201-4 PMC7605509

[57] Brown, N.B. Enhancing trust in llms: Algorithms for comparing and interpreting llms. https://arXiv.org/abs/2406.01943. (2024).

[58] Schwartz, S., Yaeli, A., & Shlomov, S. Enhancing trust in llm-based ai automation agents: New considerations and future challenges. https://arXiv.org/abs/2308.05391. (2023).

[59] Didolkar, A., Goyal, A., Ke, N.R., Guo, S., Valko, M., Lillicrap, T., Rezende, D., Bengio, Y., Mozer, M., & Arora, S. Metacognitive capabilities of llms: An exploration in mathematical problem solving. https://arXiv.org/abs/2405.12205. (2024).

[60] Belcak, P., Heinrich, G., Diao, S., Fu, Y., Dong, X., Muralidharan, S., Lin, Y.C., & Molchanov, P. Small language models are the future of agentic ai. https://arXiv.org/abs/2506.02153. (2025).

[61] Cooke, N. J. Modeling human expertise in expert systems. In *The Psychology of Expertise* (ed. Ericsson, K. A.) 29–60 (Psychology Press, 2014).

[62] Kumar, B. & Sharma, A. Examining the research on social media in business-to-business marketing with a focus on sales and the selling process. *Ind. Mark. Manage.* **102**, 122–140 (2022).

[63] Likert, R. *A technique for the measurement of attitudes* **140** (Archives of Psychology, 1932).

