# Supplementary material for: BioAgents: Bridging the gap in bioinformatics analysis with multi-agent systems
Source: Sci Rep. 2025 Nov 7;15:39036. doi: 10.1038/s41598-025-25919-z (PMC12594986; doi:10.1038/s41598-025-25919-z)
Supplement: Supplementary file 1 — Supplementary Information 1. [file 41598_2025_25919_MOESM1_ESM.pdf]

1 Supplementary Material

Our benchmarking results indicate that Phi-3 and GPT-4 perform similarly on Biostars QA pairs (Supplemental Table S1).

Model	ROUGE-1	ROUGE-2	ROUGE-L	ROUGE-L-SUM
Phi-3.5-mini	0.129	0.015	0.074	0.092
Phi-3.5-MoE	0.129	0.015	0.074	0.091
GPT-4	0.183	0.029	0.103	0.125
GPT-4o	0.122	0.014	0.072	0.091
BioAgents	0.121	0.012	0.071	0.086

Supplemental Table S1. Benchmarking on 71 Biostars Question-Answer (QA) Pairs

Outputs from BioAgents and our Human Experts for the easy and medium workflows are displayed below:

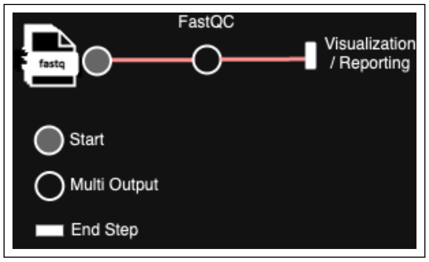

Supplemental Figure S1. Multi-Agent System on the Easy Workflow

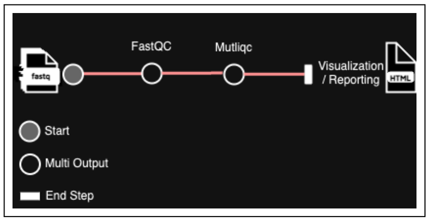

Supplemental Figure S2. Experts on Easy Workflow

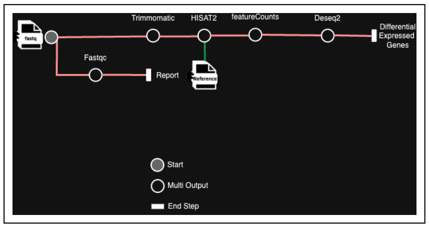

Supplemental Figure S3. Multi-Agent System on the Medium Workflow

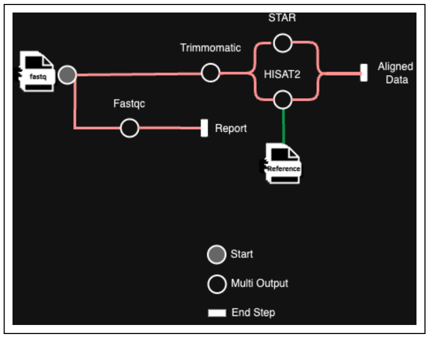

Supplemental Figure S4. Experts on Medium Workflow
